# Development, characterization, and consumer acceptance evaluation of thermally stable capsule beads containing mixed extracts of green tea and turmeric

**DOI:** 10.1038/s41598-023-46339-x

**Published:** 2023-11-07

**Authors:** Kanjana Singh, Benu Adhikari, Julia Low, Margaret Anne Brennan, Lisa Newman, Charles Stephen Brennan, Niramon Utama-ang

**Affiliations:** 1 Division of Product Development Technology, Faculty of Agro-Industry, Chiang Mai University, Chiang Mai, 50100 Thailand; 2 School of Science, RMIT University, Melbourne, VIC 3083 Australia; 3 Cluster of High Value Products From Thai Rice and Plants for Health, Faculty of Agro-Industry, Chiang Mai University, Chiang Mai, 50100 Thailand

**Keywords:** Biochemistry, Chemistry

## Abstract

The aim of this study was to investigate the ability of shell (coating) formulations comprised of alginate and glucono delta lactone (GDL) to encapsulate a mixture of green tea and turmeric extracts. Three concentrations of alginate and GDL were used at 0.5%, 0.75%, and 1%, w/v and their solid ratio was varied using a factorial design. A response surface model was applied to optimize the retention of catechin and curcuminoid contents, to determine encapsulation efficiency, and to minimize undesirable flavor and taste. Increasing the concentration of alginate and GDL significantly increased the retention of catechin and curcuminoid contents, encapsulation efficiency, and consumer acceptance (p < 0.05). The encapsulating solution containing 1% of each alginate and GDL performed the best against each criterion. The thermal treatment carried out at the boiling point of water for 15 min had a significant impact on the retention of catechin and curcuminoid content which, in the thermally-treated beads, was 5.15 and 3.85 times higher than unencapsulated, respectively. The consumer acceptance of the encapsulated beads after thermal treatment was higher than that of the unencapsulated formulations as they exhibited lesser pungent flavor and bitterness. The innovative process of thermally stable microencapsulation can produce anti-cancer activity compounds involved in functional food industrial sectors.

## Introduction

Polyphenols are increasingly used as ingredients in functional foods due to their potential benefits to human health. They are a diverse group of natural compounds produced by plants as secondary metabolites and are characterized by the presence of multiple phenol rings in their chemical structure. These phenolic rings contribute to their ability to scavenge free radicals and protect against oxidative stress, imparting antioxidant properties^1^. Polyphenols form a part of the regular human diet as they are found in fruits, vegetables, and nuts. Since polyphenols can be readily oxidized, their antioxidative potential is affected by the heat, light, and oxygen prevailing during their extraction, processing (product formulation), and storage^2^. All of these factors contribute to poor bioavailability and stability and ultimately reduce their effectiveness^3,4^. Furthermore, polyphenols have low stability in the human digestive tract and exhibit low bioavailability. When properly protected, they can be less easily rejected by the human body^5^.

One of the known methods capable of preserving the health-promoting properties of polyphenols is encapsulation, which is a process that entraps active ingredients in wall materials. Suitably carried out microencapsulation protects polyphenols and other active yet unstable micronutrients from environmental factors^6^ to preserve their stability during processing and storage, helping to prevent undesirable interactions with the food matrix^7,8^. Encapsulation can also help mask off-flavors and the undesirable taste of active compounds such as the bitterness of *Momordica charantia* Linn. extracts and the fishy and rancid aroma of rice protein extracts^9, 10^. The encapsulation of active compounds making use of the ionic gelation of natural polysaccharides is most effective^11,12^ at enhancing food product quality, protecting sensitivity to heat and ensuring stability during storage. It is used to develop microcapsules for oral delivery due to no high-temperature application encapsulation^13^. Sensitive food ingredients including polyphenols are microencapsulated using ionic gelation method to preserve their functional property from oxygen, heat, moisture, pH, etc. and to achieve control/targeted delivery^14,15^.

The encapsulating or coating materials used in the microencapsulation of active ingredients need to be effective at providing protection against stressors both inside and outside of the microcapsules^16^. Sodium alginate is one of the most used encapsulating shell materials and is known to provide efficient protection to a broad array of active compounds^17^. The encapsulation efficiency of active ingredients when alginate is used together with GDL is reported to be much higher compared to when the former is used alone^18^.

Alginate is a natural polysaccharide isolated from the cell walls of various species of brown algae (*Phaeophyceae*) and bacteria (*Azobacter vinelandii*). Alginate is broadly used in the food industry as a gelling, thickening, stabilizing and emulsifying agent. This is due to its reasonable cost and ease of use^19^. This biopolymer consists of a linear chain of (1–4) linked β-d-mannuronic acid (M) (M-block) and α-l-glucuronic acid (G) (G-block) of varying compositions and sequences^20^. The compounds to be encapsulated are dissolved in an alginate solution and then brought into contact with a second aqueous solution containing cross-linking cations (calcium solution) for gelation at room temperature^21^. The gel formation qualities depend on the nature and concentration of alginate, the concentration of chloride ions, and the solubility of the calcium ions. The addition of GDL cations is conducive to controlling the gelation process of alginate^21,22^. This ionic gelation-based encapsulation approach is very valuable in terms of preserving the stability of the phenolic components as it can be carried out at ambient temperature^11^.

Catechin from green tea extracts and curcuminoids from turmeric extracts are natural polyphenols that exhibit high antioxidant and radical scavenging activities^23,24^. Both extracts modulate cancer cell growth, metastasis, angiogenesis, and other aspects of cancer progression through different mechanisms^25–28^. Several in vitro and in vivo studies have shown that a mixture of (combined) polyphenols is more effective at inhibiting cancer growth than a single polyphenol due to the distinct inhibitory effects of various polyphenol on cells^29,30^. These findings can assist the food industry by minimizing the reliance on synthetic chemical additives and mitigating potential adverse side effects^31^. Catechin was reported to epimerize and oxidize when subjected to thermal stress (25 to 120 °C) at a neutral pH^12^. Curcuminoids have a relatively slow degradation at pH below 7 and are relatively stable following thermal degradation at temperatures of 80 °C for 2 h^32^. In addition, green tea extracts are characterized by strong bitterness and astringency^33^. Turmeric extracts are also known for their inherent pungent smell and bitter taste^16^. The encapsulation of green tea and turmeric extracts is expected to increase the stability of the polyphenols contained in both extracts, masking their undesirable smell and taste^16,33^.

In recent years, there has been an increasing interest in alginate bead encapsulation for encapsulated natural antioxidants, antimicrobials, essential oils, etc. and the non-acceptable taste of extracts before application in food products. Some researchers have studied the concentration of alginate and calcium chloride on the type of gelation on protein retention^34^, and the optimized processing conditions of natural bioactive compounds encapsulation^35,36^. Many studies have focused on the optimization concentration of single wall material as the concentration of alginate. Moreover, in food processing, the thermal process of cooking, manufacturing or food safety issues requires the thermal process for sterilization. To date, little attention has been paid to using the mixing wall materials and thermal stability of encapsulation beads. During thermal treatments, the physical properties of encapsulated beads undergo changes, however, no information on mixing wall material to enhance food product quality, stability and sensory properties can be found. In the above context, the objective of this study was to investigate the ability of encapsulating shell materials produced using alginate and GDL on green tea and turmeric extracts through the thermal process on the stability of bioactive compounds and consumer acceptance. The process of creating capsule beads was implemented at room temperature following a simple dipping procedure and passed through the sterilization process. The efficiency of the shell formulation was examined using factorial design and the response surface methodology which analyzed the remaining catechin, curcuminoid content and the consumer acceptance of encapsulated beads after the heat process as criteria. The non-heat treatment of encapsulated beads and non-encapsulated beads was used to compare as a control treatment. Finally, these encapsulated beads will be used to be involved in functional food industrial sectors.

## Methods

### Materials and chemicals

Fresh green tea leaves (*Camellia sinensis var. assamica*) were harvested on 12 June 2020 at Raming Tea Co., Ltd., Chiang Mai, Thailand. Dried rhizomes of turmeric were purchased from Premium Foods Co., Ltd., Chiang Mai province, Thailand. The food grade sodium alginate, GDL and calcium chloride were purchased from Sigma-Aldrich (St. Louis, MO, USA) with a purity ≥ 99%. Food grade of 95% ethanol was purchased from the Liquor Distillery Organization (Thailand). Catechin standards including catechin (C), epigallocatechin gallate (EGCG), epigallocatechin (EGC), epicatechin gallate (ECG), and epicatechin (EC) were purchased from Sigma-Aldrich (St. Louis, MO, USA). Curcuminoid standard was purchased from Sigma-Aldrich (St. Louis, MO, USA). Phosphoric acid, acetonitrile, tetrahydrofuran, and acetic acid were purchased from RCI Labscan Co., Ltd. (Bangkok, Thailand).

### Preparation of green tea extract

Fresh green tea leaves (*Camellia sinensis var. assamica*) were obtained from Raming Tea Co., Ltd., Chiang Mai, Thailand. The fresh green tea leaves (20 kg) were withered, roasted, rolled, dried, and milled using a hammer mill (Brook Crompton Series 2000, Huddersfield, UK) then passed through a 1.2 mm sieve. Microwave-assisted extraction was used to extract the green tea following the protocol reported in previous work^25^. Briefly, 100 g of green tea powder was extracted in 60% (v/v) of ethanol food grade (1000 mL) using microwave power at 600 W for 10 min (LG, model MS2022D, Korea). Once the extraction process was completed, the solvent was evaporated under vacuum at 40 °C using a rotary evaporator (Büchi Rotavapor R-200, Allschwil, Switzerland). The green tea extracts were dried under a freeze-dryer at − 30 °C, ending at 25 °C for 24 h using a freeze dryer with stoppering tray dryers (Labconco, USA). After that, the extracts were kept in air-tight amber bottles and stored in darkness at 4 °C for further analysis for no more than 7 days.

### Preparation of turmeric extract

Dried rhizomes of turmeric were purchased from Premium Foods Co., Ltd., Chiang Mai province, Thailand. The rhizomes was ground by a hammer mill machine (Brook Crompton Series 2000, Huddersfield, UK) and passed through a 1.0 mm sieve. We utilized the extraction process protocol described in our previous study^26^. Briefly, 5 g of turmeric powder was extracted in 95% (v/v) ethanol food grade (100 mL) using microwave-assisted extraction at 800 W for 3 min (LG, model MS2022D, Korea). The extraction was performed in cycles with 0.5 min of irradiation and 5 min of cooling time to control the temperature at 25 °C and to avoid the boiling of the solvent. Once the extraction process was completed, the solvent was evaporated under a vacuum at 40 °C using a rotary evaporator (Büchi Rotavapor R-200, Allschwil, Switzerland). The extracts were dried in a hot air oven (Binder, Germany) at 40 °C until the moisture content was less than 10%. After that, the extracts were kept in air-tight amber bottles and stored in darkness at 4 °C for further analysis for no more than 7 days.

### Preparation of capsules of mixed extracts

**Figure 1:**
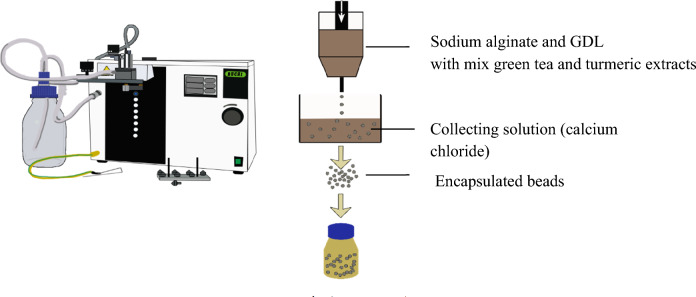
Schematic representation of the encapsulation procedure of green tea and turmeric extracts with alginate and glucono delta lactone (GDL).

A factorial design was implemented to determine the optimum concentration of the shell (coating). The concentration of alginate and GDL was varied from 0.5%, 0.75%, and 1.0% in the aqueous solution. The alginate-to-GDL ratio was varied at 0.5%, 0.75%, and 1%, w/w. The response surface model was used to monitor the effect of the alginate-to-GDL ratio on the encapsulation of green tea and turmeric extracts in retention to catechin and curcuminoid contents, encapsulation efficiency, and to minimize undesirable taste and smell. These formulations were used to encapsulate the mixture of green tea and turmeric extracts (mixed extracts) in using 10 treatments (Table 1). To obtain the encapsulated beads, alginate and GDL were initially dissolved in 100 mL of water and then 2.0 g and 1.0 g of green tea and turmeric extracts were added, adhering to the limitations of the Thai FDA. This selected ratio 2:1 of green tea and turmeric extracts is based on a previous study, which demonstrated the synergistic effect of combination extracts which has a higher potential to inhibit colon cancer compared to another ratio and single extract (In vitro studies). The solution was homogenized using a digital homogenizer (IKA, Korea) at 10,000 RPM for 5 min and drawn into a syringe with a 300 µm diameter nozzle, which was selected by the preferable size of encapsulated beads during the pilot testing with 20 participants (Encapsulator B-390, Switzerland). The pulsation unit generated mechanical vibrations and dropped the alginate solution through the nozzles into a 10% calcium chloride solution with a system frequency of 700 Hz and pressure of 14.5 kPa. These frequency, pressure and calcium alginate concentrations were varied and trialed before selection due to the completeness and strength of the beads. The beads were kept in the gelling bath to harden for 30 min, then were sieved (60 mesh) and washed with distilled water (Fig. 1). The untreated encapsulated beads were kept in a flask at ambient temperature for 24 h before analysis and the heat-treated encapsulated beads were sterilize before being kept in a flask at ambient temperature for 24 h before analysis.

**Table 1:** Operating parameters of alginate and glucono delta lactone concentration for all 10 runs carried out according to the factorial design and the responses for encapsulated green tea and turmeric extracts beads after sterilization by factorial.

Treatments	Alginate (% w/v)	GDL^2^ (% w/v)	Catechin content mg/100 g beads WB^1^	Curcuminoid content mg/100 g beads WB^1^	Catechin encapsulation efficiency (%)	Curcuminoid encapsulation efficiency (%)	Catechin and curcuminoid encapsulation efficiency (%)	Loading capacity of catechin and curcuminoid (%)
1	0.50	0.50	240.49 ± 3.99^h^	115.52 ± 4.23^h^	18.24 ± 0.30^h^	18.82 ± 0.69^i^	18.43 ± 0.86^f^	35.60 ± 0.80^e^
2	0.50	0.75	294.07 ± 4.17^ g^	119.14 ± 1.33^ g^	22.31 ± 0.31^ g^	19.41 ± 0.21^h^	21.40 ± 1.87^e^	33.06 ± 0.35^f^
3	0.50	1.00	319.60 ± 5.62^f^	129.76 ± 4.48^f^	24.25 ± 0.42^f^	21.14 ± 0.73^g^	23.26 ± 2.18d^e^	29.96 ± 0.51^g^
4	0.75	0.50	371.26 ± 2.01^e^	166.63 ± 2.54^e^	28.17 ± 0.15^e^	27.15 ± 0.41^f^	27.85 ± 0.95^d^	43.03 ± 0.14^c^
5	0.75	0.75	442.07 ± 3.11^d^	211.17 ± 2.96^d^	33.54 ± 0.23^d^	34.41 ± 0.48^e^	33.82 ± 1.74^c^	43.55 ± 0.11^c^
6	0.75	1.00	501.63 ± 2.41^c^	224.12 ± 2.34^c^	38.06 ± 0.18^c^	36.52 ± 0.38^d^	37.58 ± 2.39^b^	41.47 ± 0.25^d^
7	1.00	0.50	575.46 ± 4.26^b^	256.22 ± 3.13^b^	43.66 ± 0.32^b^	41.75 ± 0.51^c^	43.06 ± 0.34^a^	55.45 ± 0.14^a^
8	1.00	0.75	590.33 ± 4.70^a^	264.58 ± 1.97^a^	44.79 ± 0.35^a^	43.12 ± 0.32^b^	44.26 ± 0.91^a^	48.85 ± 0.38^b^
9	1.00	1.00	569.90 ± 4.28^b^	270.83 ± 4.37^b^	43.24 ± 0.32^b^	44.14 ± 0.71^a^	43.52 ± 2.59^a^	42.04 ± 0.41^d^
10	1.00	1.00	570.33 ± 2.88^b^	270.94 ± 2.59^b^	43.27 ± 0.21^b^	44.15 ± 0.42^a^	43.54 ± 1.27^a^	42.06 ± 0.20^d^

Values are the mean ± standard deviation (n = 3); ^1^WB: wet weight; ^2^GDL: Glucono delta lactone; ^a–i^represent significant differences in the same columns at (p < 0.05) within the same column.

### Determination of encapsulation efficiency

The prepared encapsulated beads from the various concentrations of alginate (0.5, 0,75, 1%) and GDL (0.5, 0,75, 1%) (10 treatments) in 100 mL of water with 2.0 g of green tea extracts (1317.80 mg of catechin content) and 1.0 g of turmeric extracts (613.58 mg of curcuminoid content) of both untreated and heat-treated samples were crushed and stirred for 12 h followed by sonication for 20 min according to Kim et al.^14^. The encapsulation efficiency, total encapsulation efficiency and loading capacity of the capsule beads were determined by the amount of catechin and curcuminoid content using Eqs. (1), (2), (3).

1 $${\text{Encapsulation efficiency }}\left( \% \right) \, = {\text{ W1}}/{\text{W2 }} \times { 1}00$$

where W1 is the amount (mg) of catechin or curcuminoid in encapsulated beads and W2 is the initial concentration of catechin content (1317.8 mg) or curcuminoid content (613.58 mg) in mixed green tea and turmeric extracts.

2 $${\text{Total encapsulation efficiency }}\left( \% \right) \, = {\text{ W1}}/{\text{W2 }} \times { 1}00$$

where W1 is the total amount (mg) of catechin and curcuminoid in the encapsulated beads and W2 is the initial concentration of catechin content (1317.8 mg) and curcuminoid content (613.58 mg) in mixed green tea and turmeric extracts.

The loading capacity of encapsulated beads was determined using Eq. (3)^37^

3 $${\text{Loading capacity }}\left( \% \right) \, = {\text{ W1}}/{\text{W2 }} \times { 1}00$$

where W1 is the amount (mg) of catechin and curcuminoid in encapsulated beads and W2 is the mass (g) of alginate and GDL.

### Scanning electron microscopy

The morphology of heat-treated encapsulated beads was examined using scanning electron microscopy (SEM) (Prisma E, thermo scientific, USA). The encapsulated beads were coated with gold and placed on the surface of the stubs and observed by SEM under an acceleration voltage of 5 kV at a magnification of 300×.

### The thermal stability of encapsulated beads

The thermal stability of the capsule beads was evaluated after the sterilization process. Briefly, 20 g capsule beads were mixed with distilled water (100 mL), the pH was adjusted (< 4.6) to keep curcuminoids in the neutral form (pH < 7)^32^ and they were packed into a glass bottle. This aqueous slurry was heated to the boiling point of water (100 °C) for 15 min and cooled down to 25 °C (sterilization). Catechin, curcuminoid content and sensory evaluation were analyzed and compared to the same statistics obtained from the untreated samples.

### Determination of catechin and curcuminoid content

Five chemical standards including catechin (C), epigallocatechin gallate (EGCG), epigallocatechin (EGC), epicatechin gallate (ECG), and epicatechin (EC) were used to determine the catechin in the beads. Catechin standards were prepared to make a stock solution of 1.0 mg/mL in 60% ethanol for each individual catechin. The stocks were stored at − 20 °C and protected from light until used. The mixed catechin standards were prepared by dilution of the stock solution to 0.05, 0.025, 0.0125, 0.00625 mg/mL in 60% ethanol. An Agilent 1200 series (Agilent Technologies, Santa Clara, CA, USA) high-performance liquid chromatography (HPLC) system with a C18 column (4.6 × 250 mm. Waters, Ireland) was used in the measurements as described in the previous study^23^. The mobile phase consisted of a mixture of elution A (phosphoric acid 86.5% v/v, 0.2% v/v in acetonitrile 12% and tetrahydrofuran 1.5% v/v) and B (73.5% phosphoric acid v/v, 0.2% v/v in 25% acetonitrile and 1.5% tetrahydrofuran) with a flow rate of 1 mL/min. The detectors were defined at wavelengths of 280 nm (detector wavelength 1) and 210 nm (detector wavelength 2) with a column temperature of 25–30 °C. A standard solution of curcuminoid was prepared by mixing curcumin (80%), demethoxycurcumin (DMC) (17%) and bisdemethoxycurcumin (BDMC) (3%). The stocks were stored at − 20 °C and protected from light until used. The curcuminoid standards were prepared by dilution of the stock solution to 0.05, 0.025, 0.0125, 0.00625 mg/mL in 95% ethanol. An Agilent 1200 series (Agilent Technologies, Santa Clara, CA, USA) high-performance liquid chromatography (HPLC) system with a C18 column (4.6 × 250 mm. Waters, Ireland) was used to examine the measurements as implemented in the previous study^26^. The mobile phase consisted of 1% (v/v) acetic acid in filtered MilliQ water (solvent A) and acetonitrile (solvent B) with a flow rate of 1 mL/min. The detector was defined at wavelengths of 425 nm.

### Consumer acceptance tests

There were two stages in the consumer acceptance test. First, was on the optimization of the wall materials of the encapsulated beads. All 50 participants between 20 and 50 years of age were asked to evaluate the consumer acceptance of the encapsulated beads, untreated (10 treatments) and heat-treated (10 treatments). Secondly, for comparison of the untreated and heat-treated encapsulated beads produced with the optimized concentration of wall materials, there was also a non-encapsulation sample (control) by 100 participants between 20 and 50 years of age. The participants were recruited from Chiang Mai University, Thailand using a balanced incomplete block design. Each stage used a different set of consumers. Potential participants were excluded if they: (1) smoked; (2) were pregnant or lactating; (3) were taking any prescription medication that may interfere with their ability to taste; or (4) had a history of food allergies that may interfere with the study. Consumers were asked to provide their demographic information and rated the acceptability of all attributes on a 7-point hedonic scale (1 = dislike extremely, 4 = neither like nor dislike, and 7 = like extremely). Both untreated and heat-treated samples were prepared by mixing 10 g of capsule beads in 100 g of distilled water. This preparation was confirmed during the pilot testing with 20 participants with the highest acceptance score. The heat-treated samples were prepared as described in the section on the thermal stability of encapsulated beads. The control sample was prepared by dissolving green tea extracts and turmeric extracts in 100 mL of distilled water at 25 °C. All consumer acceptance tests were carried out in the laboratory in which the temperature was controlled at 25 °C. The samples were sequentially presented in plastic cups (50 mL/sample), each coded with a three-digit random number, and served to the panelists. Mineral water and plain crackers were provided for rinsing the palate between samples.

### Statistical analysis

The experiments were carried out least in triplicate and the data was presented using mean ± standard deviation. A two-way analysis of variance (ANOVA) was used for the data analysis. Differences between any two mean values were significant at the 95% confidence level (p < 0.05). Analyses were performed using SPSS statistics software version 17.0. The numerical and graphical optimization techniques of the Design-Experts software (Program version 6.0.2; Stat Ease Inc, Minneapolis, USA) were used for the simultaneous optimization of the responses.

### Ethics

The study was conducted in accordance with the Declaration of Helsinki, and was approved by the Ethics Committee of Chiang Mai university Research Ethics Committee (CMUREC No.63/157; approved date 110920). The informed consent form was received as it involved human participants.

## Results and discussion

The formulation of capsule beads was standardized to maximize the potential for the protective encapsulation of catechin, curcuminoid and consumer acceptance, with the thermal stability utilizing the response surface model. A factorial design was employed to determine the composition of alginate and GDL as shell (coating) materials. The encapsulated beads were tested for catechin and its derivatives, including curcuminoid content, encapsulation efficiency, and consumer acceptance before and after the heat treatment.

### Microstructure of capsule beads

**Figure 2:**
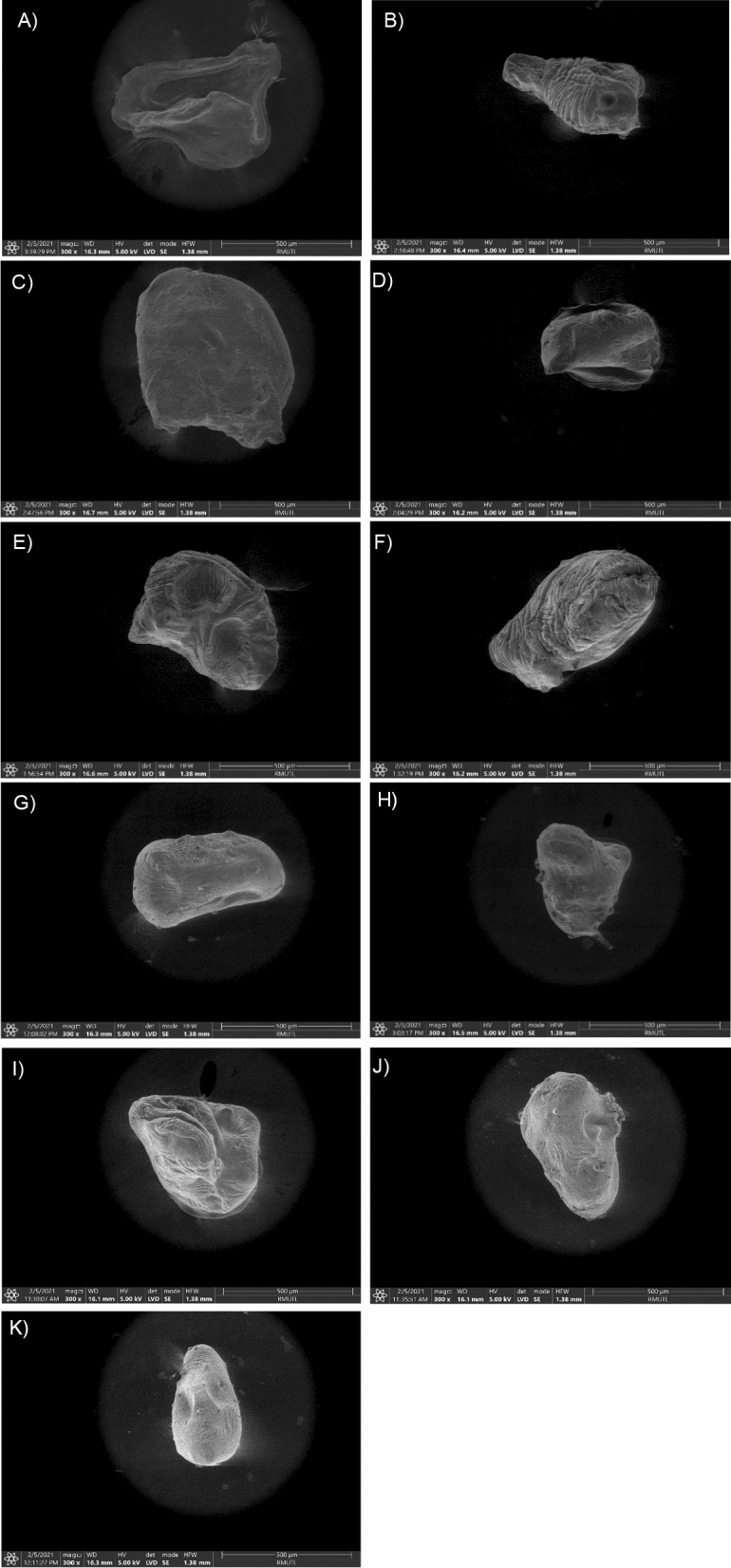
Scanning electron micrograph (SEM) of encapsulated beads after sterilization process treatment1 (**A**), treatment2 (**B**), treatment3 (**C**), treatment4 (**D**), treatment5 (**E**), treatment6 (**F**), treatment7 (**G**), treatment 8 (**H**) at a magnification ×300. Scanning electron micrograph (SEM) of encapsulated beads after sterilization process treatment (**I**), treatment (**J**) and before sterilization of treatment 10 (**K**) (optimized point) at a magnification ×300.

To better understand the influence of the composition of the shell material and concentration on the physical properties of the treated encapsulated beads, the microstructure was measured in the range of 500 microns or 0.5 mm to be observed through scanning electron microscopic (SEM) images (Fig. 2). According to the definition of microcapsule by Sonu and Goel^38^, it ranged in size from one micron (one-thousandth of an mm) to a few mm, confirming the reference to microcapsules regarding the encapsulated beads in this study. The SEM micrographs (Fig. 2A–J) unveiled that the different formulations of alginate and GDL greatly affected the morphology of the heat-treated capsule beads by showing different sizes, shapes, and different surfaces. The comparison of heat-treated and non-heat-treated (before sterilization) encapsulated beads at optimized treatments is presented in Fig. 2J–K. The heat treatment presented an alteration to the bead structure when compared to the unheated which unheated beads are homogeneous and smooth (Fig. 2K), while after heat treatment, the encapsulated beads have a greater porous structure (Fig. 2J). This reason is supported by the study of the thermal stability of calcium alginate beads by Kim et al.^12^, which found a change in bead structure from smooth to porous due to the water loss of encapsulated beads with heat treatment above 80 °C. Furthermore, when considering the different formulations of encapsulated beads after heat treatment, it was evident that increasing the concentration of alginate and GDL strengthens the structure of the resulting gels. For example, when using 0.5% alginate with 0.5% GDL as shown in Fig. 2A, utilizing 0.75% in Fig. 2E, and employing 1% in Fig. 2I,J, it is evident that the gels become progressively strength and exhibit improved capacity for substance retention in the same sequence. This observation was consistent with that of Aprilla et al.^39^ and Rajmohan and Bellmer^34^, who reported that higher alginate concentrations increased the surface tension of the beads and led to enhanced bead hardness and improved encapsulation efficiency. The capsule beads produced using treatments 1 to 6 exhibited a pronounced porous structure (Fig. 2A–F). The beads produced from treatments 7 to 10 displayed a spherical morphology characterized by a smooth and continuous surface (Fig. 2G–J). Supported by Roopa and Bhattacharya^22^, who studied the heat stability of encapsulated beads by boiling at 100 °C for 60 min which reported that the higher concentration of alginate and GDL created favorable conditions for the replacement of sodium ions by calcium ions, facilitating crosslinking with carboxylate groups and negative polar groups. This increased the interaction between the alginate and GDL chains which formed a less permeable surface, thereby impeding the diffusion of calcium ions into the interior of the beads which results in a smooth surface and increased gel strength.

### Catechin, curcuminoid content and encapsulation efficiency

The encapsulation efficiency reflects the effectiveness of the wall material composed of alginate and GDL in protecting the encapsulated mixed extracts against loss during heat treatment. It also aids in encapsulating the pungent smell and undesirable taste of the extracts. The catechin and the derivative contents in the green tea extracts and curcuminoid content in the turmeric extracts were all measured. The catechin (C), epigallocatechin gallate (EGCG), epigallocatechin (EGC), epicatechin gallate (ECG), and epicatechin (EC) contents in the green tea extracts were found to constitute 76.19, 74.75, 51.08, 60.32, and 67.11 mg/g of dry extract. The curcuminoid content in turmeric, including curcumin (CU), demethoxycurcumin (DMC), and bisdemethoxycurcumin (BDMC), was found to be 174.07, 92.85, and 39.87 mg/g, respectively of dry turmeric extract. The catechin and curcuminoid contents and encapsulation efficiency of the thermally-treated beads are presented in Table 1.

**Figure 3:**
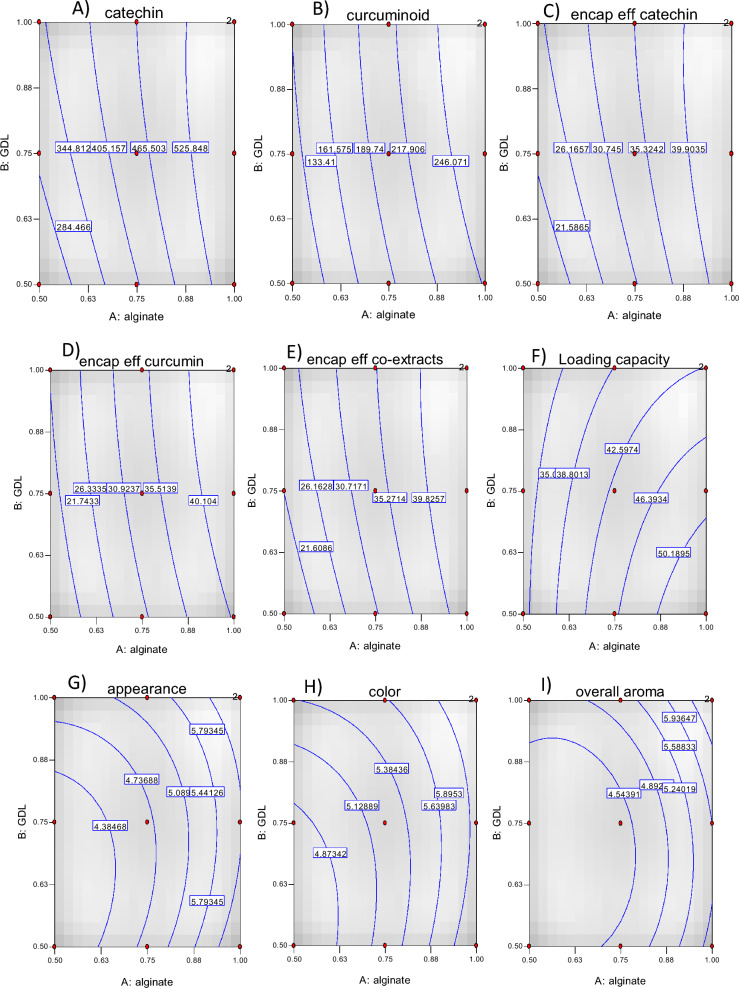

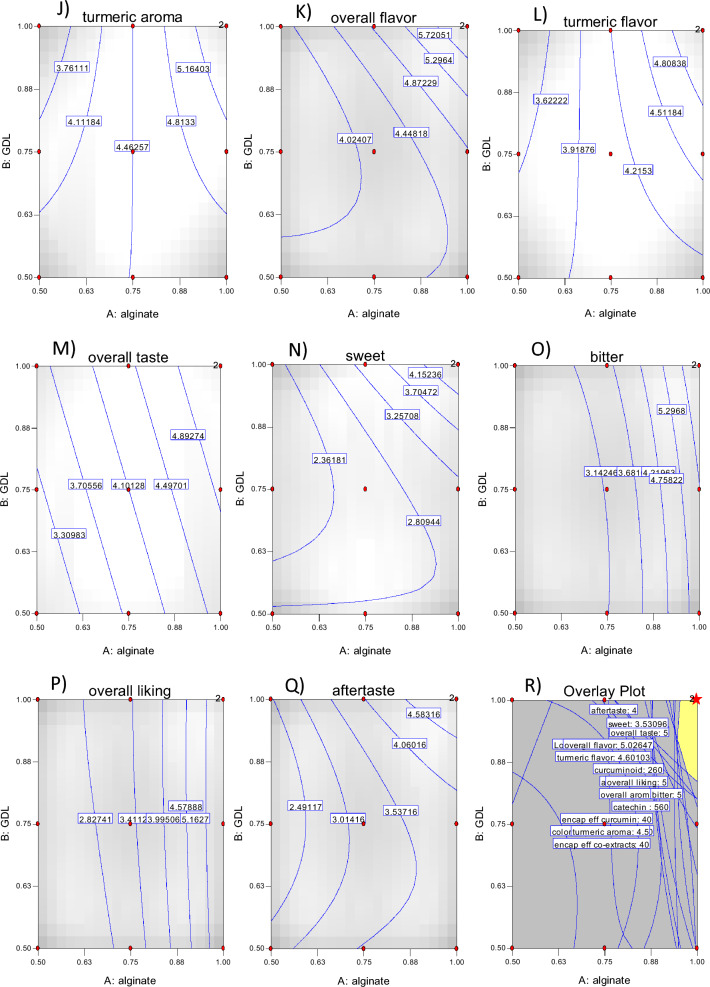
Response surface plots showing the effect of AL and GDL on catechin content (**A**), curcuminoid content (**B**), encapsulation efficiency of catechin (**C**), encapsulation efficiency of curcuminoid (**D**), encapsulation efficiency of co-extracts (**E**), Loading capacity (**F**), Appearance (**G**), color (**H**), overall aroma (**I**), turmeric aroma (**J**), overall flavor (**K**), turmeric flavor (**L**), overall taste (**M**), sweet (**N**), bitter (**O**), overall liking (**P**), aftertaste (**Q**) and response surface plots of optimized condition (**R**) red star: optimum point.

The total catechin and curcuminoid content in the capsule beads ranged from 240.49 to 590.33 mg/100 g and 115.52 to 270.94 mg/100 g of encapsulated beads, respectively, and they were found to be significantly different. As shown in Tables 1, 2 and Fig. 3A,B, an increase in alginate and GDL concentration significantly increased the catechin and curcuminoid content. The coefficient of determination (R^2^) between the experimental and predicted data was 0.95, confirming the suitability of the response surface model to explain the relationship between the variables^16^. The encapsulation of the mixed extracts was influenced by the structure of the wall (coating) and the nature of the junctions (crosslinks) formed in the bead structure. Wongverawattanakul et al.^40^ observed that concentration of alginate affected the encapsulation efficiency of the extracts through the formation of junctions formed in the presence of calcium chloride, a divalent ion, which trapped the bioactive compounds inside the beads. Shamsudin et al.^41^ noted that GDL contributed to the formation of a milder acidic environment, enhancing the crosslinking process in the presence of a calcium chloride solution. Other studies have highlighted that the encapsulation method, the cross-linking process, and the type of encapsulating materials used had an affect on the encapsulation of bioactive compounds^42–44^.

**Table 2:** Regression equations for the responses affected by alginate and glucono delta lactone concentration and evaluation parameters: coefficients of determination (adj-R^2^). ANOVA results: *p*-value and lack of fit.

Factor	Regression coefficient (β)									
	Constant (β_0_)	Linear		Quadratic		Interaction	R^2^	F	*p*-value	Lack of fit
		β_1_	β_2_	β_11_	β_12_	β_22_				
Catechin content	447.65	145.36	32.43	− 8.24	− 23.51	− 23.51	0.9555	36.69	0.0017	0.0069
Curcuminoid content	203.98	70.65	13.84	− 8.51	− 0.73	− 0.73	0.9539	38.23	0.0018	0.0036
Catechin encapsulation efficiency (%)	33.97	11.03	2.46	− 0.63	− 1.78	− 1.78	0.9550	36.69	0.0017	0.0069
Curcuminoid encapsulation efficiency (%)	33.24	11.51	2.26	1.39	− 0.12	− 0.12	0.9539	38.22	0.0018	0.0037
Catechin and curcuminoid encapsulation efficiency (%)	33.74	11.18	2.39	− 0.87	− 1.26	− 1.26	0.9576	41.68	0.0015	0.0036
Loading capacity of catechin and curcuminoid (%)	43.13	7.85	− 3.54	− 1.96	− 0.67	− 2.10	0.9547	16.84	0.0086	0.0051
Appearance	4.68	0.81	0.21	0.28	− 0.21	− 0.21	0.8132	8.83	0.0277	ns
Color	5.24	0.57	0.19	0.14	− 0.19	− 0.19	0.9426	30.58	0.0028	ns
Overall aroma	4.45	0.67	0.22	0.47	0.05	0.05	0.9074	18.63	0.0071	ns
Turmeric aroma	4.47	0.58	− 0.02	–	0.47	0.47	0.6951	7.84	0.0169	ns
Overall flavor	4.13	0.61	0.28	0.11	0.51	0.51	0.9536	38.03	0.0018	ns
Turmeric flavor	4.10	0.52	0.12	–	0.37	0.37	0.9213	36.12	0.0003	ns
Overall taste	4.10	0.85	0.34	–	–	–	0.8240	22.07	0.0009	ns
Sweet	2.57	0.60	0.25	0.58	–	–	0.6880	5.96	0.0384	ns
Bitter	3.19	1.33	0.23	0.76	0.17	0.17	0.9770	77.49	0.0005	ns
Overall liking	3.31	1.60	0.13	0.83	− 0.10	− 0.10	0.9635	48.50	0.0011	ns
Aftertaste	3.18	0.93	0.23	0.27	0.40	0.40	0.8365	10.21	0.0214	ns

β_0_: intercept; β_1_: alginate concentration (%); β_2_: glucono delta lactone concentration (%); β_11_: alginate concentration (%) x alginate concentration (%); β_12_: alginate concentration (%) x glucono delta lactone concentration (%); β_22_: glucono delta lactone concentration (%) x glucono delta lactone concentration (%).

The total encapsulation efficiencies, as well as the individual encapsulation efficiencies of the catechin and curcuminoids, are presented in Table 1. The total encapsulation efficiency ranged from 18.43 to 44.26%. The individual encapsulation efficiency of the catechin and curcuminoids fell within the range of 18.24% to 44.79% and 18.82% to 44.15%, respectively. The statistical analysis (Table 2) indicated that the model was deemed to be suitable as there was no significant lack of fit for both total encapsulation efficiency and individual encapsulation efficiency. The variation of the alginate and GDL concentration in the solution had a significant (p < 0.05) impact on both total and individual efficiency values as indicated by the coefficient of determination (R^2^) varying between 0.95 to 0.96. An increase in alginate concentration led to a significant increase in the encapsulation efficiency of catechin, curcumin, and catechin with curcumin, similar to the effect observed with an increase in GDL concentration (Table 2, Fig. 3C–E). The different concentrations of AL and GDL used in this study had significant differences in encapsulation efficiency (p < 0.05). These findings are consistent with the observations of Najafi-Soulari et al.^45^ who reported that the concentration of alginate influenced the encapsulation efficiency of the lemon balm extracts. Liu et al.^46^ and Najafi-Soulari et al.^45^ reported that the increase in alginate and guluronic acid content in the solution increased the interaction with the calcium ions which was conducive to the formation of hydrogel network. We observed that the encapsulation process also impacted the loss of encapsulated mix extracted from the beads.

**Figure 4:**
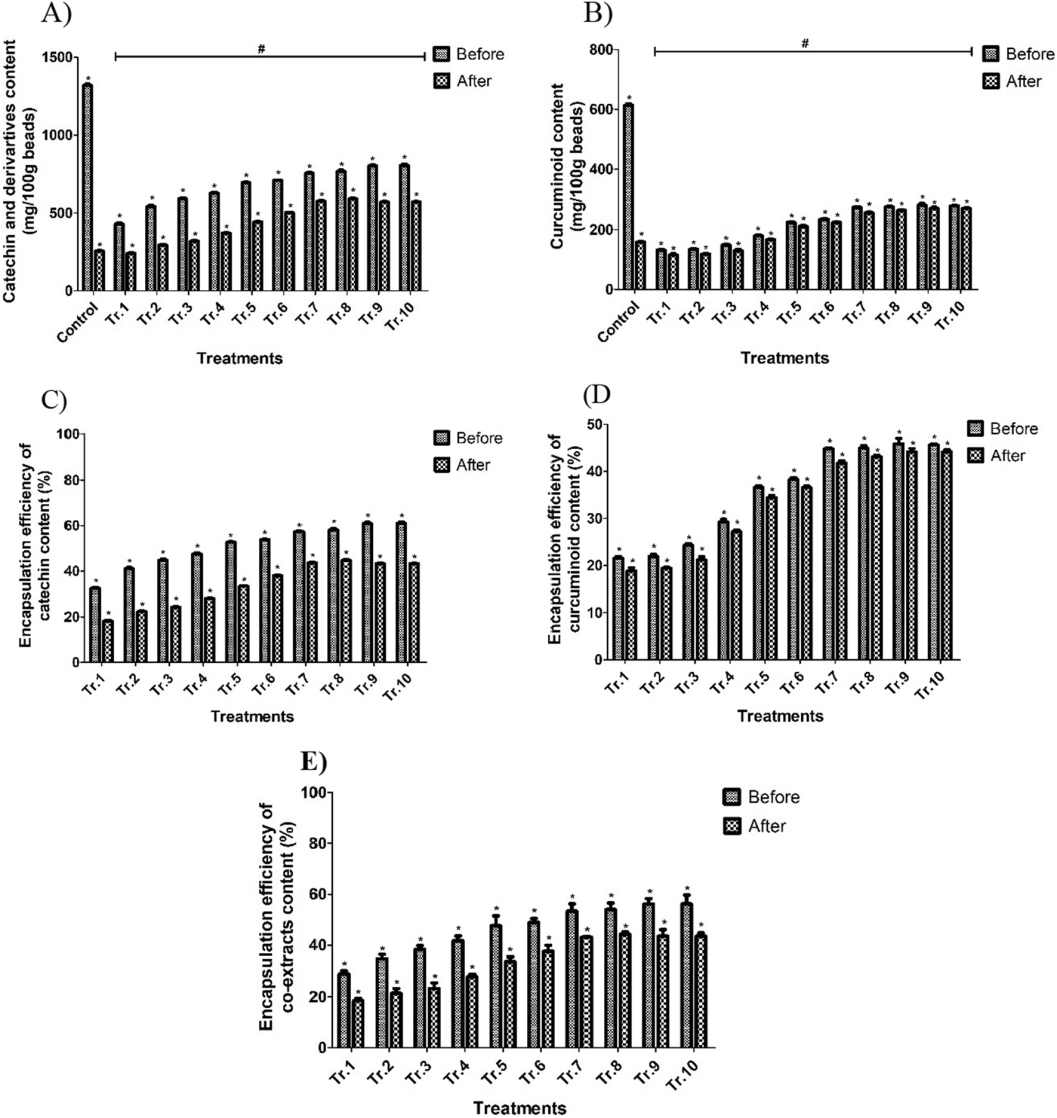
The comparison of catechin content (**A**), curcuminoid content (**B**), encapsulation efficiency of catechin content (**C**), encapsulation efficiency of curcuminoid content (**D**) and encapsulation efficiency of co-extracts (**E**) of encapsulated beads before and after sterilization with different concentration of AL and GDL *(p < 0.05) and the comparison of catechin content (**A**), curcuminoid content (**B**) and encapsulation efficiency (**E**) of non-encapsulated beads after sterilization (control) with 10 runs of encapsulated beads after sterilization *(p < 0.05).

Figure 4 presents a comparison of the catechin and curcuminoid content in encapsulated beads from 10 different treatments, with the starting concentration of the alginate solution containing the mixed extracts serving as the control. The results show that the encapsulated beads contained lower levels of bioactive compounds compared to the control. The starting concentration of the alginate solution contained 1317.80 mg of catechin and 613.58 mg of curcuminoid, respectively. The highest total encapsulation efficiency achieved was 44.26%. This resulted in encapsulated beads containing 590.33 mg of catechin and 264.58 mg of curcuminoid content, respectively. The loss of catechin and curcuminoid from the mixed extracts could be attributed to the bead generation process where some of the extract was lost in the calcium chloride solution. Deladino et al.^47^ reported that a loss of extracts occurred in the encapsulation process, particularly during the immersion of alginate beads in the calcium chloride. To mitigate the loss of bioactive compounds during bead generation, it is important to determine the optimum concentration of wall material and cross-linking ions in the bead generation bath^48^. While a positive correlation between alginate concentration and encapsulation efficiency was observed in this study, several studies have reported a decrease in encapsulation efficiency when an excessive alginate concentration were used. Soliman et al.^49^ reported that the encapsulation efficiency of essential oil decreased when the concentration of alginate increased above 2% w/v. This decrease was attributed to the formation of smaller-sized pores, resulting in a lower amount of extract being entrapped within the polymeric shell matrix. This is the reason why we have limited the upper concentration of alginate at 1% (w/v). According to Belscak-Cvitanovic et al.^43^, the encapsulation efficiency of plant extracts in the calcium-alginate gel matrix typically falls within 10% to 20%. This method involves the diffusion of gelling ions into the alginate dispersion. As the concentration of alginate solution increases, the viscosity increases sharply and reduces the diffusion of calcium ions into the interior of the beads. This approach also comes with the drawback of producing an inhomogeneous gel due to its rapid gel setting process^22^. To address this issue, sodium alginate is used together with a sequestering agent (e.g. GDL) so then calcium ions are released gradually, helping to form a more homogeneous gel^50^. GDL forms a slow-release acid solution and lowers the pH of the aqueous phase, leading to the gradual release of calcium cations from the calcium chloride. The released cations then cross-link with the alginate chains, facilitating gel formation^51^.

### Stability of capsule beads during heat treatment

In the food industry, various heat treatments are applied to prepare food products. Heat treatment are necessary to ensure food safety and also to ensure there is desirable texture, flavor and taste. Ching et al.^52^ highlighted the use of alginate gels to enhance the physical properties, quality, and stability of food products during storage. In this study, the stability of the encapsulated beads containing mixed extracts was evaluated at an acidic pH (< 4.6) at the boiling point of water for 15 min. This combination of pH and temperature–time regime is similar to the sterilization regimes applied to high-acid foods (e.g. fruit juices)^53^. The catechin and curcuminoids contents were compared in terms of stability to the heat-treated samples. Non-encapsulated extracts were used as a control for comparison purposes.

#### Stability of catechin and curcuminoid content of capsule beads

Figure 4A presents a comparison of the catechin content between untreated and heat-treated capsule beads from 10 different treatments and the control. Prior to the heat process, the catechin content of all treatments ranged from 428.71 to 805.03 mg/100 g. After heat treatment, a significant decrease (p < 0.05) in catechin content was observed across all treatments, ranging from 240.49 to 590.33 mg/100 g. The curcuminoid content also showed a significant decrease after heat treatment. The curcuminoid content of the untreated encapsulated beads ranged from 131.83 to 281.60 mg/100 g, while the treated beads showed a decreased content ranging from 115.52 to 270.94 mg/100 g (Fig. 4B). Farahani et al.^54^ reported that a heat treatment akin to pasteurization (72 °C, 10 min) applied to beverages enriched with beads impacted the structure and pores of the calcium alginate beads, leading to the release of a portion of the encapsulated extract into the aqueous medium. The catechin and curcuminoid content of the control treatment (non-encapsulation) is presented in Fig. 4A,B, respectively. The heating process decreased the catechin and curcuminoid content much more severely. The catechin content reduced from 1317.80 to 344.03 mg, representing a reduction of 73.89% and the curcuminoid content decreased from 613.58 to 404.73 mg, corresponding to a decrease of 34.03%. Furthermore, the catechin content and curcuminoid content of all encapsulated beads within the heat-treated samples showed significant differences compared to the corresponding value of the control treatment. The comparison of the bioactive compounds between the untreated and heat-treated encapsulated beads is presented in Fig. 4A,B, enabling the observation that the heat treatment significantly decreased the catechin and curcuminoid content in all treatments. Despite the observed loss of bioactive compounds during the heat process in encapsulated beads, the higher retention of the compounds in treatment 4 to 10 was still observed compared to the non-encapsulated beads that were heat treated (Fig. 4A,B). Therefore, the encapsulation process using alginate-GDL beads showed great promise due to effectively encapsulating polyphenol-rich extracts of green tea and turmeric, while also imparting better stability under boiling conditions compared to non-encapsulation (control).

#### Encapsulation efficiency of capsule beads

The heating process resulted in a loss of encapsulation efficiency in the mixed extracts in all treatments of the encapsulated beads. The individual and total encapsulation efficiency values were significantly affected by the heating process, primarily due to the decrease in catechin and curcuminoid content. The encapsulation efficiency of catechin decreased from 32.53–61.09% to18.24–44.79% (Fig. 4C). The encapsulation efficiency of the curcuminoids also decreased from 21.48–45.89% to 18.82–44.15% (Fig. 4D). The encapsulation efficiency of the mixed extracts, before and after the heat treatment, ranged from 29.02 to 56.17% and 18.43 to 44.26% (Fig. 4E), respectively. The loss of catechin was higher than the loss of curcuminoid content upon heat treatment. The higher heat-induced loss of catechin could be attributed to epimerization and oxidation under thermal stress at elevated temperatures as was used in this study^55^. On the other hand, curcuminoids are shown to have a relatively slow degradation and are relatively stable during thermal degradation at temperatures of 80 °C for 2 h^32^. Several studies have reported that alginate encapsulation can effectively reduce the loss of bioactive compounds during thermal treatments. As described above, the loss of catechin and curcuminoids from encapsulated beads was significantly lower compared to the loss of the unencapsulated extracts under an identical thermal treatment^12,56^. Sun-Waterhouse et al.^57^ and Vargas et al.^17^ observed that the physical properties of alginate beads underwent changes during heat treatment. At higher temperatures, the beads exhibited a much more porous structure due to the loss of water which caused a greater loss of encapsulated compounds. The loading capacity of heat-treated encapsulated beads, shown in Table 1, an increase in alginate and GDL concentration leads to a decrease in loading capacity, as shown in the regression model in Table 2 and Fig. 3F. These results agree with those reported by Essifi et al.^58^ who found that increasing the alginate polymer concentration causes a decrease in the loading capacity with a rise in encapsulation efficiency. Similar results were reported^59,60^, the results also show that increasing the alginate polymer concentration causes a rise in encapsulation efficiency and a decrease in the loading capacity. The obtained micro-beads with increasing the concentration of the polymer can be explained based on the higher approaching of the alginate chains and subsequently decreasing the pores sizes forming within alginate matrices, resulting in squeezing the gel micro-beads leading to a decrease of the loading capacity^58^. The encapsulated beads produced using 1% alginate and 1% GDL demonstrated the lowest percentage loss of catechin as well as curcuminoid. This higher retention could be attributed to the increase in alginate concentration which promoted a greater cross-linking with calcium ions in the presence of (1%, w/v) GDL. Additionally, the slow-release acid solution of GDL enhanced the cross-linking of alginate chains with calcium ions, leading to the formation of a more uniform gel^22,46^. This formulation was able to better maintain the structural integrity and strength of the gel, restricting the mobility of any water.

### Consumer acceptance

**Figure 5:**
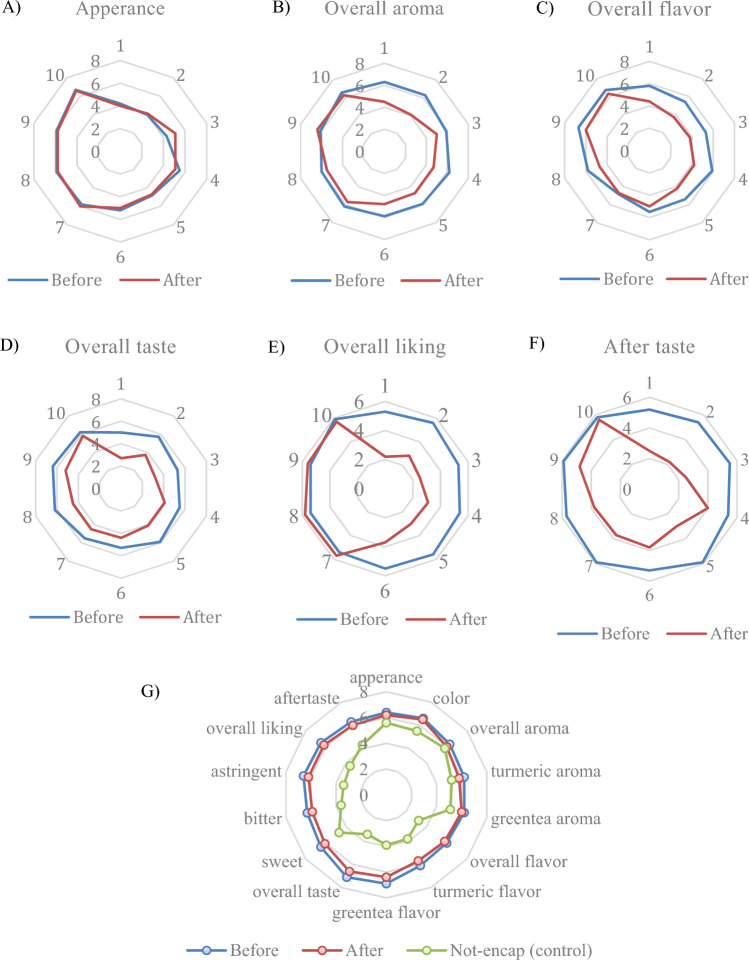
The comparison of hedonic score of sensory attributes of encapsulated alginate beads before and after sterilization with different concentration of AL and GDL (**A**–**F**) and encapsulated beads using AL and GDL at optimum point before, after and non-encapsulation (control) (**G**).

Ionic gelation-based encapsulation is a process that can be utilized to entrap active ingredients within a wall material, thereby preserving their stability during thermal processing and storage. This method effectively prevents undesirable interactions occurring between the active compounds present in the core and food matrix^8^. Green tea extracts are known for their strong bitterness and astringency^33^ and turmeric extracts possess a characteristic pungent smell^16^. Thus, the encapsulation shell matrix developed in this work is expected to mask the off-flavor and taste associated with these compounds, enhancing their palatability and consumer appeal^9^. The results of the sensory evaluation indicated that the preference score of heat-treated encapsulated beads of the mixed extracts was influenced by the concentrations of alginate and GDL. Both the single and interaction factor of the alginate and GDL concentrations had an impact on the sensory properties (Tables 2 and S1). Increasing the concentration of alginate and GDL significantly enhanced the appearance, color, overall aroma, turmeric aroma, overall flavor, turmeric flavor, overall taste, sweetness, bitterness, overall liking, and aftertaste (Fig. 3G–Q). The coefficient of determination (R^2^) ranged from 0.80 to 0.98, indicating a high level of correlation. Among the different formulations, the use of 1% alginate and 1% GDL resulted in higher preference rating scores (Fig. 5A–F) due to the masking effect of the shell material. It is commonly accepted that encapsulation, when used as part of a properly developed encapsulation process, can reduce the release of undesirable flavor and mask an undesirable taste.

Szente et al.^61^ highlighted the fact that off-flavors and off-tastes are intensified due to processing, which can be masked through encapsulation. The consumer acceptance data of both untreated and heat-treated encapsulated beads is compared in Fig. 5G. The sensory scores for all attributes (overall aroma, flavor, taste, liking, and aftertaste) slightly decreased after heat treatment. For example, in treatment 2, the overall flavor decreased from 5.4 to 3.7. However, there were no significant differences (p > 0.05) in the scores of these attributes between the beads before and after sterilization in treatments 9 and 10. An increase in both AL and GDL concentrations was found to enhance the preference scores. The higher gel strength achieved with higher concentrations of alginate and GDL played an important role in controlling the release of the mixed extracts into water, improving the sensory taste. Figure 5G shows the sensory score comparison between non-encapsulated and optimized untreated and heat-treated capsule beads. The preference scores for all attributes were lower in the non-encapsulated treatment compared to the encapsulated beads in terms of overall flavor, taste, bitterness, astringency, and liking. Slightly different preference scores were observed between the encapsulated beads before and after the heat treatment; specifically, the scores after the heat treatment were lower. The utilization of this facile dipping (extrusion)-based encapsulation process with an optimal concentration of shell (coating) material for encapsulating combined extracts that are rich in phenolic compounds also helps to improve consumer preference.

### Optimal conditions and verification

In the food industry, it is important to minimize the changes in foods during thermal treatment processes such as pasteurization and sterilization. In this context, the optimum concentration of shell (coating) materials, namely alginate and GDL, was determined to achieve a high encapsulation efficiency, minimum loss of catechin and curcuminoid, and maximum consumer acceptance of the encapsulated beads. All response values obtained from the encapsulated bead formulations achieved the desired characteristics. The optimal encapsulating solution formulation was, as shown in Fig. 3R, a blend of 1% (w/v) alginate and 1% (w/v) GDL as it demonstrated the best performance. The verified experimental values for all parameters of the selected model were close to the (response surface) model’s predicted values. The experimental values for each of the parameters obtained using this encapsulating shell for catechin content, curcuminoid content, encapsulation efficiency of catechin, encapsulation efficiency of curcuminoid, encapsulation efficiency of co-extracts and sensory score of appearance, color, overall aroma, turmeric aroma, overall flavor, turmeric flavor, overall taste, sweet, bitter, overall liking, and aftertaste were 576.32 ± 2.84 mg/100 g, 270.23 ± 1.89 mg/100 g, 43.73 ± 4.01%, 44.04 ± 3.17%, 43.03 ± 4.34%, 6.4 ± 1.3, 6.6 ± 0.6, 6.3 ± 1.0, 6.2 ± 0.9, 6.0 ± 1.5, 6.1 ± 1.2, 7.1 ± 1.2, 6.5 ± 0.7, 6.3 ± 0.8, 6.5 ± 0.7, 6.3 ± 0.9, respectively. The experimental errors between the experimental values and (response surface) predicted values for these 16 parameters ranged from 1.87% to 9.63% indicating that the fitted model for the encapsulation of mixed extracts was satisfactory and reliable.

## Conclusion

A factorial design for the response surface method was applied to determine the optimal concentration of shell materials namely alginate and GDL at 0.5%, 0.75%, and 1%, w/v for the encapsulation of mixed extracts of green tea and turmeric in capsule beads, as well as to assess their stability during thermal treatment. The ability to encapsulate shell materials on green tea and turmeric extracts successfully encapsulated the bioactive compounds and non-acceptable taste of extracts through the thermal process. The results demonstrated that increasing the alginate and GDL concentrations in their blend solution significantly (p-value < 0.05) improved the encapsulation efficiency (R^2^; 0.95), catechin content (R^2^; 0.95), curcuminoid content (R^2^;0.95), and consumer acceptance (R^2^ > 0.68), of the encapsulated mixed extracts**.** The encapsulating (coating) solution containing 1% (w/v) alginate and 1% (w/v) GDL was identified as the optimal formulation, achieving the highest encapsulation efficiency, superior heat stability, and favorable consumer reception. The catechin and curcuminoid content retention in thermally treated beads was 5.15 and 3.85 times higher than unencapsulated. Therefore, this simple dipping encapsulation process with an optimal concentration of shell material for encapsulating combined extracts rich in bioactive compounds also helps improve consumer preference compared to non-encapsulation mixing extracts. As a result of the thermal stability of bioactive compounds in optimized microcapsules through the thermal process, this microencapsulation process has become increasingly relevant to the food industry. Considering that microcapsules that contain antioxidant activity and are improved by preventing the possible bitter taste of the extracts from being used directly can be involved in functional food industrial sectors.

### Supplementary Information

Supplementary Table S1.

## Data Availability

The datasets used and/or analyzed during the current study are available from the corresponding author on reasonable request.
