# Supplementary material for: Development, characterization, and consumer acceptance evaluation of thermally stable capsule beads containing mixed extracts of green tea and turmeric
Source: Sci Rep. 2023 Nov 7;13:19299. doi: 10.1038/s41598-023-46339-x (PMC10630281; doi:10.1038/s41598-023-46339-x)
Supplement: Supplementary file 1 — Supplementary Table S1. [file 41598_2023_46339_MOESM1_ESM.docx]

**Supplementary Table**

**Table S1** Operating parameters of alginate and glucono delta lactone concentration for all 10 runs carried out according to the factorial design and the hedonic score of sensory attributes of encapsulated green tea and turmeric extracts beads after sterilization ^1^.

| **TR** | **AL** | **GDL** | **Appearance** | **Color** | **Overall aroma** | **Turmeric aroma** | **Green tea aroma**  **(ns)** | **Overall flavor** | **Turmeric flavor** | **Green tea flavor**  **(ns)** |
| --- | --- | --- | --- | --- | --- | --- | --- | --- | --- | --- |
| 1 | 0.50 | 0.5 | 4.0±1.4^f^ | 4.7±1.1^e^ | 4.5±1.2^e^ | 4.0±1.3^g^ | 5.1±1.2 | 4.4±1.2^e^ | 3.8±1.2^d^ | 4.1±1.1 |
| 2 | 0.50 | 0.75 | 4.1±1.2^f^ | 4.7±1.2^e^ | 4.1±1.3^f^ | 4.1±0.9^fg^ | 4.4±1.2 | 3.7±1.4^g^ | 3.5±1.0^e^ | 4.0±1.2 |
| 3 | 0.50 | 1.00 | 5.1±1.6^d^ | 5.4±1.4^c^ | 5.0±1.5^d^ | 3.5±1.2^h^ | 3.5±1.3 | 3.8±1.3^g^ | 3.4±1.1^e^ | 3.4±1.1 |
| 4 | 0.75 | 0.50 | 5.1±1.5^d^ | 5.2±1.5^d^ | 4.7±0.9^e^ | 5.1±1.1^c^ | 4.1±1.2 | 4.2±1.1^f^ | 4.0±1.5^c^ | 4.4±0.8 |
| 5 | 0.75 | 0.75 | 4.7±1.1^e^ | 5.2±1.3^d^ | 4.7±1.4^e^ | 4.2±1.5^f^ | 3.7±1.4 | 4.2±1.2^f^ | 4.4±1.2^b^ | 4.2±1.4 |
| 6 | 0.75 | 1.00 | 5.0±0.9^d^ | 5.7±1.1^b^ | 4.8±1.7^e^ | 4.2±0.8^f^ | 4.1±1.5 | 5.0±1.4^c^ | 4.0±1.4^c^ | 5.0±1.2 |
| 7 | 1.00 | 0.50 | 6.0±1.2^a^ | 6.1±1.2^a^ | 5.7±1.4^b^ | 4.5±1.3^e^ | 4.7±1.5 | 4.7±0.9^d^ | 4.1±0.8^c^ | 4.1±1.5 |
| 8 | 1.00 | 0.75 | 5.8±1.3^b^ | 6.1±1.1^a^ | 5.5±0.8^c^ | 4.7±1.2^d^ | 4.5±1.4 | 4.7±1.2^d^ | 4.5±1.2^b^ | 4.4±1.1 |
| 9 | 1.00 | 1.00 | 5.8±1.5^b^ | 6.2±0.8^a^ | 6.4±1.1^a^ | 5.5±1.1^b^ | 5.8±1.1 | 6.0±1.1^ab^ | 5.1±1.1^a^ | 4.8±1.4 |
| 10 | 1.00 | 1.00 | 6.6±1.6^a^ | 6.0±1.0^a^ | 6.3±1.2^a^ | 5.9±1.4^a^ | 5.1±1.3 | 6.3±1.0^a^ | 5.2±0.9^a^ | 6.7±1.3 |

^1^ Values are the mean ± standard deviation (n = 50); ^a–g^ represent significant differences in the same columns at p < 0.05; ns: not significant (p > 0.05) within the same column; TR: treatment; Al: alginate; GDL: glucono delta lactone.

**Table S1** **(continued)** Operating parameters of alginate and glucono delta lactone concentration for all 10 runs carried out according to the factorial design and the hedonic score of sensory attributes of encapsulated green tea and turmeric extracts beads after sterilization ^1^.

| **TR** | **AL** | **GDL** | **Overall taste** | **Sweet** | **Bitter** | **Astringent**  **(ns)** | **Overall liking** | **After taste** | **Amount of bead**  **(ns)** | **Bead texture**  **(ns)** |
| --- | --- | --- | --- | --- | --- | --- | --- | --- | --- | --- |
| 1 | 0.50 | 0.5 | 2.7±1.0^g^ | 2.8±1.0^e^ | 2.8±1.2^f^ | 3.2±0.8 | 2.2±1.1^g^ | 2.5±1.2^f^ | 5.0±1.2 | 4.6±1.2 |
| 2 | 0.50 | 0.75 | 3.7±1.2^e^ | 2.2±1.3^g^ | 2.4±1.3^i^ | 3.1±1.0 | 2.8±1.2^e^ | 2.2±1.3^g^ | 5.2±1.4 | 5.0±1.1 |
| 3 | 0.50 | 1.00 | 3.2±1.2^f^ | 2.5±1.2^f^ | 3.0±1.2^i^ | 3.2±1.5 | 2.5±1.6^f^ | 2.5±1.1^f^ | 5.2±1.5 | 4.0±1.3 |
| 4 | 0.75 | 0.50 | 4.1±1.2^d^ | 3.1±1.2^e^ | 3.1±1.0^e^ | 3.2±1.1 | 3.1±1.6^d^ | 4.0±1.2^c^ | 5.0±1.2 | 5.3±1.5 |
| 5 | 0.75 | 0.75 | 4.1±1.4^d^ | 2.1±1.5^g^ | 3.4±1.4^d^ | 3.2±1.4 | 3.0±1.0^d^ | 3.0±1.4^e^ | 5.0±1.1 | 4.0±1.2 |
| 6 | 0.75 | 1.00 | 4.4±1.5^c^ | 2.8±1.6^e^ | 3.4±1.4^d^ | 3.7±1.3 | 3.7±0.8^c^ | 3.8±1.6^d^ | 5.4±1.3 | 4.0±1.0 |
| 7 | 1.00 | 0.50 | 4.5±1.3^c^ | 2.8±1.2^de^ | 5.0±1.6^c^ | 3.0±1.2 | 5.7±1.4^ab^ | 3.7±1.1^d^ | 5.0±1.4 | 4.2±1.2 |
| 8 | 1.00 | 0.75 | 4.5±1.4^c^ | 3.4±1.0^c^ | 5.3±1.7^b^ | 2.8±1.1 | 5.8±1.3^a^ | 3.8±1.0^d^ | 4.7±1.4 | 5.0±0.9 |
| 9 | 1.00 | 1.00 | 5.2±1.6^b^ | 4.2±1.0^b^ | 5.8±1.5^a^ | 4.2±1.1 | 5.6±1.1^b^ | 4.8±1.0^b^ | 5.1±1.0 | 5.2±1.3 |
| 10 | 1.00 | 1.00 | 5.8±1.3^a^ | 5.3±1.2^a^ | 5.9±1.3^a^ | 5.6±0.9 | 5.7±1.3^ab^ | 5.6±1.1^a^ | 5.3±1.1 | 4.5±1.2 |

^1^ Values are the mean ± standard deviation (n = 50); ^a–g^ represent significant differences in the same columns at p < 0.05; ns: not significant (p > 0.05) within the same column; TR: treatment; Al: alginate; GDL: glucono delta lactone.
